# Overcoming evasive resistance from vascular endothelial growth factor a inhibition in sarcomas by genetic or pharmacologic targeting of hypoxia-inducible factor 1α

**DOI:** 10.1002/ijc.27666

**Published:** 2012-06-26

**Authors:** Yeo-Jung Kim, Hae-June Lee, Tae-Min Kim, TS Karin Eisinger-Mathason, Alexia Y Zhang, Benjamin Schmidt, Daniel L Karl, Michael S Nakazawa, Peter J Park, M Celeste Simon, Sam S Yoon

**Affiliations:** 1 Department of Cancer Biology, University of Pennsylvania School of MedicinePhiladelphia, PA; 2 Abramson Family Cancer Research Institute, University of Pennsylvania School of MedicinePhiladelphia, PA; 3 Department of Surgery, Massachusetts General HospitalBoston, MA; 4 Harvard Medical SchoolBoston, MA; 5 Division of Radiation Effects, Korea Institute of Radiological and Medical SciencesSeoul, Korea; 6 Center for Biomedical Informatics, Harvard Medical SchoolBoston, MA; 7 Howard Hughes Medical Institute, University of Pennsylvania School of MedicinePhiladelphia, PA

**Keywords:** sarcomas, hypoxia, HIF-1α, VEGF-A

## Abstract

Increased levels of hypoxia and hypoxia-inducible factor 1α (HIF-1α) in human sarcomas correlate with tumor progression and radiation resistance. Prolonged antiangiogenic therapy of tumors not only delays tumor growth but may also increase hypoxia and HIF-1α activity. In our recent clinical trial, treatment with the vascular endothelial growth factor A (VEGF-A) antibody, bevacizumab, followed by a combination of bevacizumab and radiation led to near complete necrosis in nearly half of sarcomas. Gene Set Enrichment Analysis of microarrays from pretreatment biopsies found that the Gene Ontology category “Response to hypoxia” was upregulated in poor responders and that the hierarchical clustering based on 140 hypoxia-responsive genes reliably separated poor responders from good responders. The most commonly used chemotherapeutic drug for sarcomas, doxorubicin (Dox), was recently found to block HIF-1α binding to DNA at low metronomic doses. In four sarcoma cell lines, HIF-1α shRNA or Dox at low concentrations blocked HIF-1α induction of *VEGF*-A by 84–97% and *carbonic anhydrase 9* by 83–93%. HT1080 sarcoma xenografts had increased hypoxia and/or HIF-1α activity with increasing tumor size and with anti-VEGF receptor antibody (DC101) treatment. Combining DC101 with HIF-1α shRNA or metronomic Dox had a synergistic effect in suppressing growth of HT1080 xenografts, at least in part *via* induction of tumor endothelial cell apoptosis. In conclusion, sarcomas respond to increased hypoxia by expressing HIF-1α target genes that may promote resistance to antiangiogenic and other therapies. HIF-1α inhibition blocks this evasive resistance and augments destruction of the tumor vasculature.

**What’s new?:**

Despite their initial promise, anti-angiogenic therapies have been a disappointment in the clinic. One reason is that solid tumors often become resistant to these drugs. Tumors that respond poorly to this type of therapy have increased activation of the hypoxia-induced transcription factor HIF-1α which can enhance tumor survival and progression. In this study, the authors report that this evasive resistance can be overcome by adding low-dose doxorubicin or shRNA to inhibit HIF-1α activity. They are thus developing a clinical trial combining the angiogenesis inhibitor bevacizumab with metronomic doxorubicin in sarcoma patients.

Soft tissue sarcomas arise in nearly 10,000 persons in the United States each year, striking individuals of all ages (median age of 50 years), with roughly 40% of patients dying of either locoregional recurrence or distant metastasis.^1^ The treatment of primary tumors usually includes surgery and radiation, and sometimes chemotherapy. Local recurrence after aggressive surgery alone can be as high as 33% for extremity tumors and 82% for retroperitoneal tumors.^2^, ^3^ Radiation therapy has been prospectively demonstrated to decrease local recurrence for extremity and truncal tumors,^2^, ^4^ and retrospective studies suggest that radiation therapy can reduce local recurrence for retroperitoneal and pelvic tumors.^5^, ^6^ Despite aggressive surgery and radiation, sarcomas adjacent to vital structures (*e.g.*, major vessels and nerves) and all retroperitoneal and pelvic tumors still have a significant risk of local recurrence. Furthermore, up to 50% of patients with large, high-grade sarcomas develop distant metastases, most frequently to the lung.^7^ The benefit of adjuvant chemotherapy in preventing local and distant recurrence is modest at best.^8^

It is now well established that regions within solid tumors including sarcomas experience mild to severe hypoxia owing to aberrant vascular function.^9^ The oxygen diffusion limit from blood vessels is about 145 μm, and thus, new tumor vasculature or co-option of existing vessels is required for tumors to grow beyond a microscopic size.^10^ As tumors expand, there exists a critical balance between tumor angiogenesis, or new blood vessel formation, and hypoxia. Tumor cells respond to hypoxic stress through multiple mechanisms, including stabilization of hypoxia-inducible factor 1α (HIF-1α).^11^ Stabilized HIF-α is then transported to the nucleus, where it forms a dimer with the constitutively expressed aryl hydrocarbon receptor nuclear translocator (ARNT) subunit. HIF dimers then bind hypoxia-responsive element DNA sequences and consequently activate expression of at least 150 genes whose products orchestrate adaptive responses including those mediating tumor angiogenesis *e.g.*, *vascular endothelial growth factor A (VEGF-A)*,^9^ invasion (*e.g.*, c-*Met*),^12^ cellular metabolism [*e.g.*, *carbonic anhydrase 9 (CA9)*],^13^ and metastasis (*e.g.*, *FOXM1*).^14^, ^15^ Agents targeting HIF-1α are in various stages of clinical development,^16^ and the most commonly used chemotherapeutic drug for sarcomas, doxorubicin (Dox), was recently found to block HIF-1α binding to DNA at low metronomic doses.^17^

VEGF-A is likely the most important factor driving tumor angiogenesis.^18^ We have previously shown that HIF-1α upregulates the expression of VEGF-A in sarcomas,^19^ and circulating levels of VEGF-A are elevated on average tenfold in patients with sarcoma compared to controls.^20^ The expression of VEGF-A in sarcomas correlates with extent of disease and survival.^21^ Inhibition of VEGF-A or its receptors can effectively suppress tumor angiogenesis in mouse sarcoma models,^19^, ^22^ and numerous anti-VEGF agents are in various phases of clinical trials or approved for patients with cancer.^23^

The effect of VEGF-A inhibition on intratumoral hypoxia and HIF-1α activity may vary between different tumors. Tumor blood vessels are immature, dilated, tortuous and highly permeable with erratic flow,^24^, ^25^ and many of these abnormalities can be attributed to the overexpression of VEGF-A.^26^ These characteristics led to areas of hypoxia in tumors. Administration of anti-VEGF agents can result in reduced vessel irregularity, diameter and permeability and can transiently improve the delivery of oxygen.^27^ However, sustained anti-VEGF therapy can ultimately lead to loss of tumor vessels and increased hypoxia.^28^ There has been recent significant controversy on the effects of VEGF inhibition on primary tumor invasiveness and metastatic potential.^29^ Casanovas and coworkers^30^, ^31^ found that VEGFR-2 inhibition of *RIP1-Tag2* mouse pancreatic endocrine tumors led to increased intratumoral hypoxia along with increased tumor invasiveness and liver metastases, and Ebos *et al*.^32^ found that sunitinib (which targets VEGF and other pathways) increased liver and lung metastases for both experimental and spontaneous metastases. This conflicts with other preclinical studies showing inhibition of metastases with VEGF inhibitors^33^ as well as clinical studies demonstrating that bevacizumab as single-agent therapies can prolong patient survival against metastatic renal cell cancer and other cancers.^34^, ^35^ The effects of VEGF inhibition in primary sarcomas on hypoxia, HIF-1α activity and HIF-related phenotypes such as tumor progression, metastasis and radiation response are currently unknown.

We recently completed a Phase II clinical trial of neoadjuvant bevacizumab and radiation therapy for patients with resectable soft tissue sarcomas.^36^ Twenty patients with intermediate- or high-grade soft tissue sarcomas of ≥5 cm in size received bevacizumab (an anti-VEGF-A antibody) for 2 weeks followed by 6 weeks of bevacizumab combined with radiation therapy (50 Gy). Tumor tissue samples were obtained before treatment and 10 days after the start of bevacizumab. Bevacizumab and radiation resulted in a good response (defined as ≥80% pathologic necrosis) in nine of 20 tumors (45%), which is over double the historical response rate seen with radiation alone. High initial microvessel density (MVD; τ = 0.53, *p* = 0.0031) and decrease in MVD after bevacizumab alone (τ = 0.43, *p* = 0.0154) significantly correlated with a good response to the combination of bevacizumab and radiation. As part of this clinical trial, gene expression microarray data were obtained on tumor samples prior to the start of treatment. Tumors with a good response *versus* poor response to combination therapy with bevacizumab and radiation were distinguished by a 24-gene signature that included *PLAUR* (plasminogen activator, urokinase receptor), a gene which is transcriptionally regulated by HIF-1α.^36^

In our study, further analysis of gene expression microarrays from this clinical trial suggested that a strong HIF-1α transcriptional program in sarcomas may contribute to treatment resistance and progression. Thus, we analyzed anti-VEGF treatment and HIF-1α inhibition in sarcoma cell lines *in vitro* as well as in a sarcoma mouse model and demonstrated the therapeutic potential of this novel strategy.

## Material and Methods

### Microarray analysis

Tumor samples were obtained from a Phase II clinical trial of neoadjuvant bevacizumab and radiation therapy for resectable soft tissue sarcomas as previously described.^36^ RNA was isolated from tumor tissue using the Qiagen RNeasy kit (Qiagen, Valencia, CA). RNA quality was assessed using 2100 Bioanalyzer (Agilent, Palo Alto, CA), and amplification was performed using the Illumina TotalPrep RNA Amplification Kit (Illumina, San Diego, CA). Amplified cRNAs were hybridized on HumanRef-8 Expression BeadChips (Illumina), which targets more than 24,000 known genes. Image analysis was carried out using Illumina’s BeadStudio v3.0.14 Gene Expression Module. All statistical analyses were conducted using the statistical software R (http://www.r-project.org).

The supervised hierarchical clustering of 140 genes transcriptionally regulated by HIF-1α was performed using 1 − *r* (Pearson’s correlation) as a distance metric with a complete linkage. Gene Set Enrichment Analysis (GSEA) was used to identify the Gene Ontology (GO) functional categories with significantly different expression between good and poor responders.^37^ GO categories were obtained from MSigDB (c5 GO category; http://www.broadinstitute.org/gsea/msigdb/index.jsp). The significance of enrichment was measured by phenotypic label permutation. Microarray data have been uploaded in Gene Expression Omnibus (GEO) (GEO submission #GSE31715).

### Cell lines

MS4515 mouse pleomorphic undifferentiated sarcoma cells and MS5907 mouse pleomorphic undifferentiated sarcoma cells were derived from genetically engineered mouse models of sarcoma (*LSL-Kras^G12D/+^*/*Trp53^fl/fl^* and *LSL-Kras^G12D^*^/+^; *Ink4A/Arf^fl/fl^*), which we have previously described.^22^, ^38^ HT1080 human fibrosarcoma cells, SKLMS-1 human leiomyosarcoma cells and DC101 hybridoma cells were obtained from the American Type Culture Collection (ATCC). Human umbilical vein endothelial cells (HUVECs) and human dermal microvascular endothelial cells (HDMECs) were obtained from Lonza (Basel, Switzerland). All endothelial cells were used within eight passages. Cancer cell lines were actively passaged for less than 6 months from the time that they were received from ATCC or the NCI Tumor Repository, and the UKCCCR guidelines were followed.^39^

All human and mouse sarcoma cell lines were maintained in Dulbecco modified Eagle medium supplemented with 10% fetal bovine serum, 100 U/ml penicillin, 100 μg/ml streptomycin and 2 mM l-glutamine. All endothelial cells were grown in EGM-2-MV media (Lonza).

DC101 antibody was produced from DC101 hybridoma cells using the BD CELLine 1000 system (BD Biosciences, San Jose, CA) following the manufacturer’s instructions or purchased from BioXCell (West Lebanon, NH). Dox was purchased from Teva Pharmaceuticals (Petah Tivka, Israel).

### *In vitro* assays

Cell proliferation and migration were determined as previously described.^40^ In brief to determine cell proliferation, equal numbers of cells were plated in 24-well plates and incubated for 16 hr under normoxia (21% O_2_) or hypoxia (0.5% O_2_) under the specified conditions. Cell number was then determined using a thiazolyl blue tetrazolium bromide (MTT; Sigma, St. Louis, MO) assay, with optical density read at 550 nm with a reference wavelength of 650 nm. To determine cell migration assay, equal numbers of cells were placed in a modified Boyden chamber under normoxia or hypoxia under the specified conditions for 4–18 hr. Nonmotile cells were removed from the top of the chamber insert using a cotton swap. Cells were then washed with PBS, fixed in methanol, permeabilized with 0.1% Triton-X 100 (Sigma) and stained with DAPI (Invitrogen, Carlsbad, CA). Cells were imaged using an inverted Olympus IX81 fluorescence microscope using Slidemaker software (Geeknet, Fairfax, VA). Cells were counted using ImageJ software (NIH).

### shRNA

Silencing of *HIF1-α* was achieved *via* lentiviral transduction of the following specific shRNA vectors obtained from Santa Cruz Biotechnology (Santa Cruz, CA): human *HIF-1α* shRNA sc-35561, mouse *HIF-1α* shRNA sc35562 and scramble shRNA control sc-108080. Maximal knockdown of *HIF1α* occurred 72- to 96-hr post-transduction.

### Western blot analysis

For Western blot analysis of HIF-1α, cells were incubated in 21% or 0.5% oxygen for 24 hr. Samples were collected in RIPA buffer (Sigma) containing Complete Protease Inhibitor Cocktail (Roche Diagnostics, Indianapolis, IN), and protein concentration was determined by BSA assay (Biorad, Hercules, CA). Western blot analysis was performed for HIF-1α using the following antibodies: HIF-1α (C-Term) Polyclonal Antibody (1:1,000, cat. no. 10006421; Cayman Chemical, Ann Arbor, MI) and GAPDH (1:1,000, cat. no. G9545; Sigma).

### Quantitative RT-PCR

For analysis of mRNA expression, cells were incubated in 21% or 0.5% oxygen for 24 hr. RNA was isolated from cell lines using Trizol (Invitrogen) following the manufacturer’s instructions. Total RNA was isolated from tumor tissue preserved in RNA Later using RNeasy Mini Kit (Qiagen) following the manufacturer’s instruction. RNA concentration was determined by Nanodrop 1000 (Thermoscientific, West Palm Beach, FL). cDNA was synthesized using the Superscript First-Strand Synthesis System (Invitrogen) with random hexamers. Quantitative real-time PCR analysis was performed using the 7900HT Fast Real-Time PCR System (Applied Biosystems, Foster City, CA) using 100 ng of cDNA product and Syber Green PCR Master mix (Applied Biosystems), per manufacturer’s instructions. Primers for human genes were as follows: *VEGF-A* (for) 5′-GAATGCAGACCAAAGAAAGAC-3′, (rev) 5′-GTGGTGACATGGTTAATCGG-3′; *CA9* (for) GGTTTGGATGTATCTGCACTG-3′, (rev) 5′-GAATTCACATGGACTGGCTC-3′; *FOXM1* (for) 5′-AATCGGGTTAAGGTTGAGGAG-3′, (rev) 5′-GAGAAAGGTTGTGACGAATAGAG-3′; *GAPDH* (for) 5′-CCCCTTCATTGAC CTCAACTACA-3′, (rev) 5′-CGCTCCTGGAGGATGGTGAT-3′. Primers for mouse genes were as follows: *VEGF-A* (for) 5′-GAATGCAGACCAAAGAAAGAC-3′, (rev) 5′-GTGGTGACATGGTTAATCGG-3′; *CA9* (for) 5′-GGTTTGGATGTATCTGCACTG-3′, (rev) 5′-GAATTCACATGGACTGGCTC-3′; *FOXM1* (for) 5′-AATCGGGTTAAGGTTGAGGAG-3′, (rev) 5′-GAGAAAGGTTGTGACGAATAGAG-3′; *GAPDH* (for) 5′-CCCCTTCATTGACCT CAACTACA-3′, (rev) 5′-CGCTCCTGGAGGATGGTGAT-3′.

### Enzyme-linked immunosorbent assays

For analysis of secreted VEGF-A protein, conditioned media was collected after 24-hr incubation. Secreted human and mouse VEGF-A level was measured using the following commercially available enzyme-linked immunosorbent assay (ELISA) kits: Human VEGF-A Duoset and mouse VEGF-A Duoset (all from R&D Systems, Minneapolis, MN). Manufacturer’s protocols were followed, and samples were measured in duplicate. Mean values were used as the final concentration. ELISA plates were read using the Emax Precision Microplate Reader (Molecular Devices, Sunnyvale, CA). Cell population was measured by MTT assay. Optical density values from the result of ELISA were divided into the optical density from MTT assay to normalize for cell number.

### Hypoxyprobe and necrosis

Hypoxia in tumors was measured using the Hypoxyprobe™-1 kit (HPI, Burlington, MA) following manufacturer’s instructions. Standard hematoxylin and eosin (H&E) staining was also performed on tissue sections. Images from each section were stitched together in Adobe Photoshop to create a large scan image of the whole section. Areas of necrosis (based on H&E sections) and areas of hypoxia (based on Hypoxyprobe™-1 staining) were quantified using ImageJ Software.

### Immunohistochemical and immunofluorescence microscopy

HIF-1α immunohistochemistry of paraffin-embedded sections was performed as previously described.^41^ HIF-1α was stained using mouse anti-HIF-1α Ab-4 mAb (1:10,000, NB100-105; Novus, Littleton, CO). Sections were counterstained with hematoxylin. The number of HIF-1α-positive nuclei was quantified by image analysis using software ImageJ (http://rsb.info.nih.gov/ij). HIF-1α-positive brown nuclei and HIF-1α-negative blue nuclei were counted in five random photographed fields for each tumor.

CD31 immunohistochemical localization and analysis of MVD were performed as previously described.^42^ For detection of EC apoptosis, TUNEL assay and CD31 immunofluorescence were performed. Paraffin-embedded sections were deparaffinized and incubated with goat anti-CD31 Ab (1:100, SC-1506; Santa Cruz Biotechnology) overnight at 4°C. Following washing, sections were incubated with rabbit anti-goat Alexa 594-conjugated secondary antibody (1:500, A11080; Molecular Probes, Carlsbad, CA) for 1 hr at room temperature. Then, sections were treated with the DeadEnd Fluorometric TUNEL system (Promega, Madison, WI) to detect apoptosis, following the manufacturer’s instruction. Subsequently, sections were stained with DAPI (0.2 μg/ml) for 3 min. Images were obtained on a Zeiss microscope and analyzed using AxioVison 4.0 software (Carl Zeiss, Thornwood, NY).

### Mouse studies

All mouse protocols were approved by the Massachusetts General Hospital Subcommittee on Research Animal Care. To generate subcutaneous flank tumor, 10^6^ HT1080 cells were resuspended in 100 μl of Hank’s balanced salt solution and injected subcutaneously into the right flank of athymic nude mice following xylazine/ketamine anesthesia. Six mice were used for each group. Tumors were measured three times per week for a maximum of 3 weeks, and tumor volume (TV) was calculated by using the following formula: TV = length × (width)^2^ × 0.52. Treatment began when the TVs were ∼50 mm^3^, and DC101 (400 μg per mouse), isotype control IgG_1_s (40 μg per mouse) and/or Dox (1.0 mg/kg) were injected intraperitoneally three times a week.

### Statistical analysis

Groups were compared using GraphPad 3.10 software (InStat). *p* values were calculated using Student’s *t*-test. For comparisons between more than two groups, treatment groups were compared to the control group using one-way ANOVA with Bonferroni adjustment for multiple comparisons. *p* values < 0.05 were considered significant.

## Results

**Figure 1:**
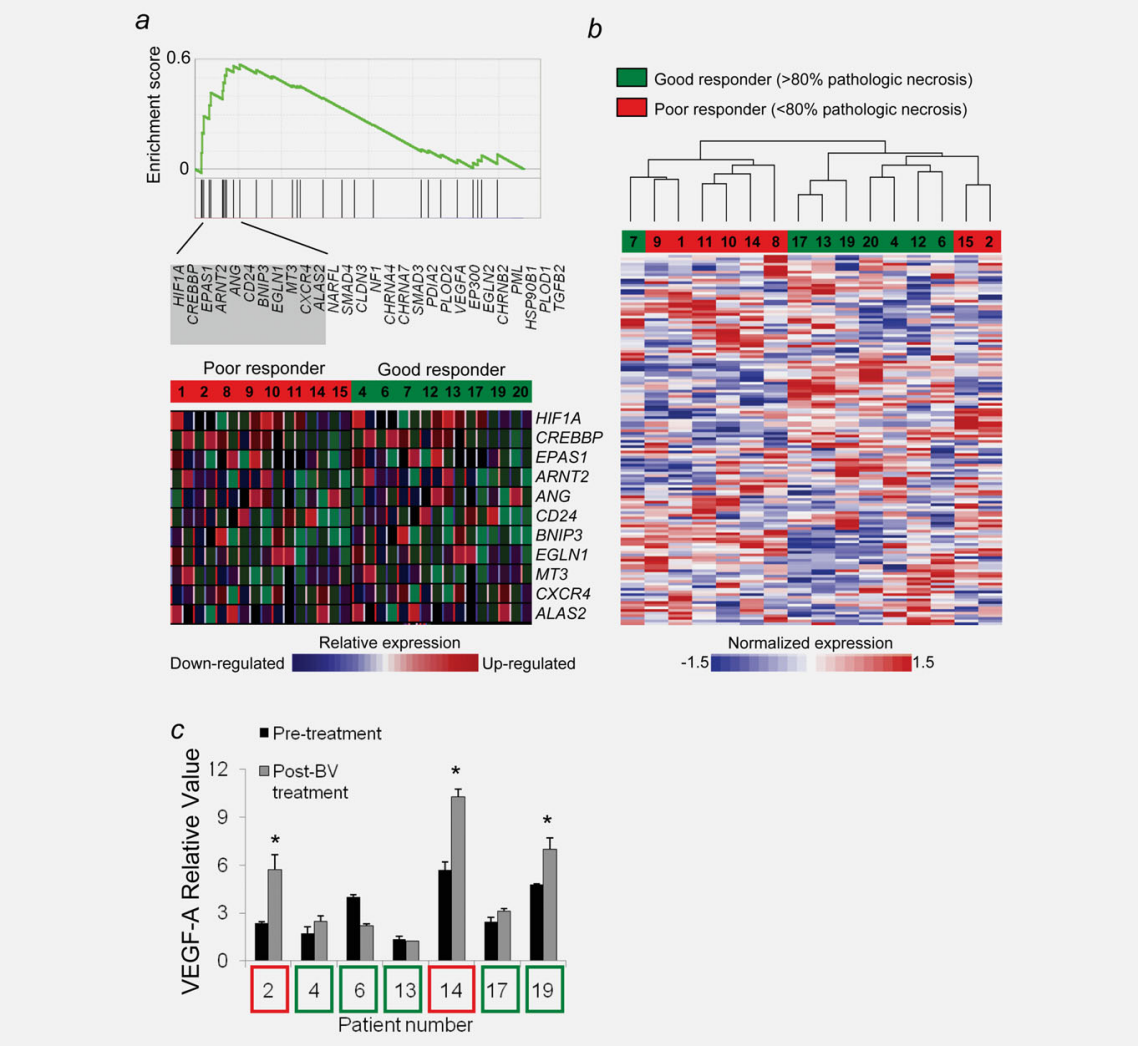
(*a*) A Gene Set Enrichment Analysis plot of the “Response to hypoxia” category from Gene Ontology is shown (top). Genes (*x*-axis) are ordered by their *t*-statistics comparing poor and good responders. The upregulated and downregulated genes in poor responders are placed on the left and right, respectively. The enrichment score (*y*-axis) is a cumulative sum reflecting the degree of over-representation for the genes in this category compared to the rest of the genes; a high enrichment score indicates the presence of hypoxia-related genes among the genes that are significantly different in the good/poor responder phenotype. The locations of the 28 hypoxia-related genes are shown in the middle bar, with the gene symbols for the 11 genes that contribute to the maximum value of the enrichment score shown in gray. The relative expression levels of the 11 genes are also shown in a heat map (bottom). Red and blue represent the relative upregulation and downregulation, respectively, compared to its average expression. (*b*) Supervised hierarchical clustering analysis of 140 hypoxia-responsive genes. Poor and good responders are indicated by red and green, respectively. This analysis demonstrates that the vast majority of good and poor responders can be differentiated based on the expression of hypoxia-related genes. Dendogram is shown at top. (*c*) Relative mRNA levels of *VEGF-A* in sarcomas before and after bevacizumab (BV) treatment. Relative value is in relation to the lowest level of expression, which was assigned a value of 1. Poor and good responders are indicated by red and green boxes, respectively. Bars represent standard deviation. *p < 0.05 compared to pretreatment level.

From the Phase II clinical trial of neoadjuvant bevacizumab and radiation for sarcoma described above, we had gene expression microarray data from tumors prior to the start of treatment.^36^ We explored the differential gene expression between tumors with a subsequent good pathological response (≥80% necrosis) *versus* poor pathological response (<80% necrosis) to bevacizumab and radiation using GSEA of 1,454 GO categories. Overall, 14 and 18 GO categories showed significant (False discovery rate < 0.1) upregulation in poor and good responders, respectively (Supporting Information Table 1). The GO category “Response to hypoxia,” which contains 28 genes, was upregulated in tumors with a poor response to bevacizumab and radiation (nominal *p* value = 0.08; Fig. 1*a*). One of the genes that most highly contributed to the identification of the enrichment of this GO category set was HIF-1α. Thus, we performed a supervised hierarchical clustering analysis of 140 genes transcriptionally regulated by HIF-1α (Supporting Information Table 2) and found that these genes reliably clustered tumors into those with a good *versus* poor response to bevacizumab and radiation (Fig. 1*b*). Paired biopsy specimens taken before and after the start of bevacizumab were available for seven patients, five of which had a good response to treatment and two of which had a poor response. In the two tumors that had a poor response to bevacizumab and radiation, quantitative RT-PCR for *VEGF-A*, a HIF-1α target gene, demonstrated significant upregulation (Fig. 1*c*). These data suggest that a strong HIF-1α-mediated transcriptional program in sarcomas may contribute to treatment resistance and tumor progression. This analysis led to the underlying hypothesis for our study: VEGF-A and HIF-1α play critical and interdependent roles in regulating sarcoma progression.

**Figure 2:**
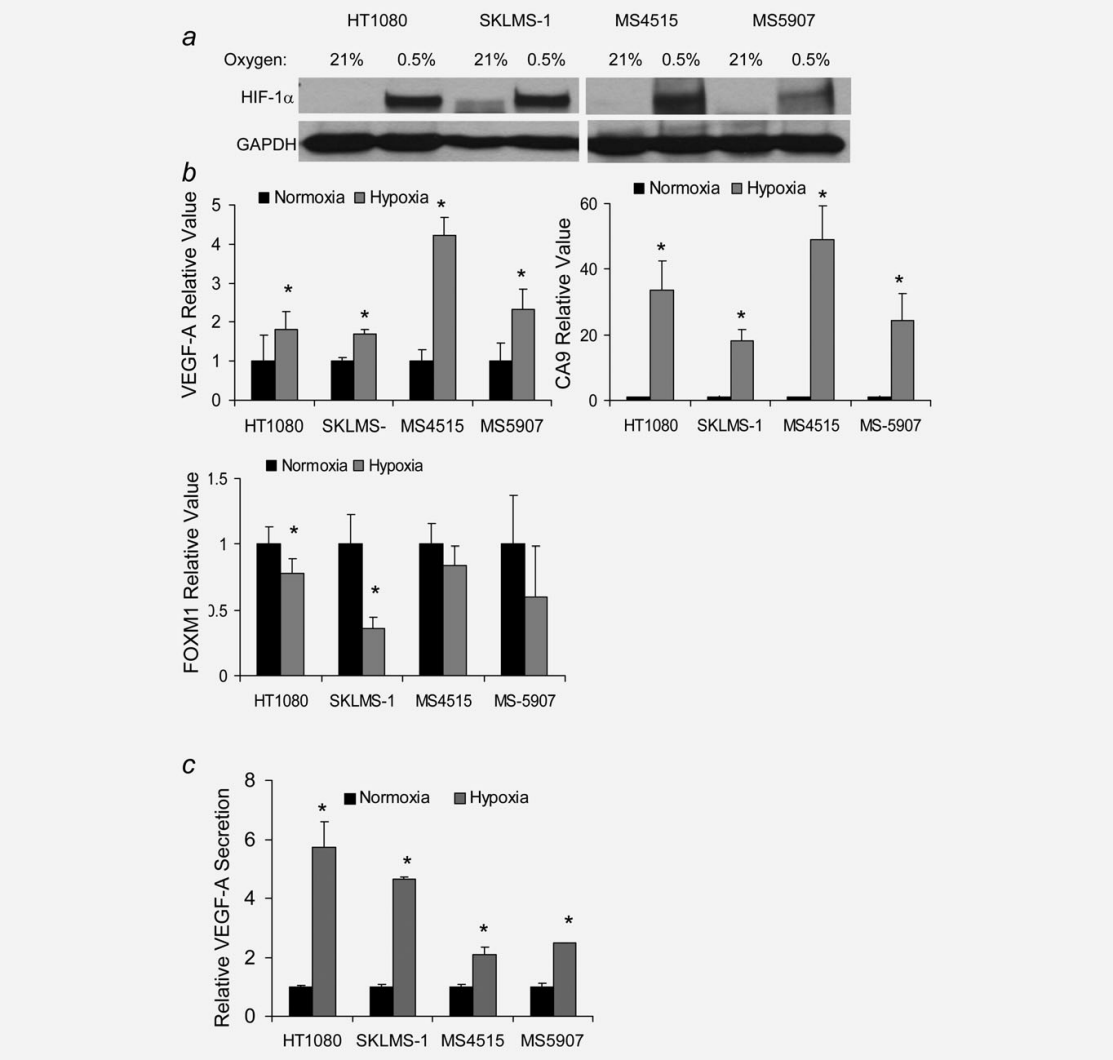
(*a*) Western blot analysis of HIF-1α in human and mouse sarcoma cell lines in 21% oxygen (normoxia) and 0.5% oxygen (hypoxia). GAPDH blot serves as loading control. (*b*) Relative mRNA levels of *VEGF-A*, *CA9* and *FOXM1* in human and mouse sarcoma cell lines under normoxic and hypoxic conditions. (*c*) VEGF-A protein secreted into media of human and sarcoma cell lines under normoxic and hypoxic conditions. For relative value, expression level under normoxia for each cell line assigned a value of 1. Bars represent standard deviation. *p < 0.05 compared to normoxia level.

To better determine the role of HIF-1α in various sarcomas, we next examined HIF-1α and HIF-1α target genes in four sarcoma cell lines, two of human origin (HT1080 human fibrosarcoma and SKLMS human leiomyosarcoma) and two of mouse origin (MS4515 and MS5907). HIF-1α levels were upregulated in all four cell lines in response to 0.5% O_2_ (Fig. 2*a*). We examined the levels of HIF-1α target genes associated with angiogenesis (*VEGF-A*), metabolism (*CA9*) and metastasis (*FOXM1*) by qRT-PCR (Fig. 2*b*). *VEGF-A* was upregulated in all four cell lines under hypoxia by 1.7- to 2.4-fold. In the human sarcoma cell lines, *CA9* was upregulated 18- to 36-fold, whereas *FOXM1* remained relatively unchanged or slightly decreased. In the mouse sarcoma cell lines, *CA9* was upregulated 24- to 49-fold, whereas *FOXM1* levels again remained relatively unchanged or decreased slightly. We confirmed changes in VEGF-A at the protein level by measuring the secretion of VEGF-A from these cell lines under hypoxia (Fig. 2*c*). VEGF-A protein secretion increased 2.1- to 5.7-fold in all cell lines following exposure to hypoxia. Of note, the differences in upregulation of *VEGF-A* mRNA *versus* VEGF-A protein under hypoxic conditions may be related to inherent differences in the cell lines in their response to hypoxia, differences in translation of *VEGF-A* mRNA or differences in secretion or degradation of VEGF-A protein. Thus, sarcoma cell lines respond to hypoxic stress by upregulating the expression of certain HIF-1α target genes.

**Figure 3:**
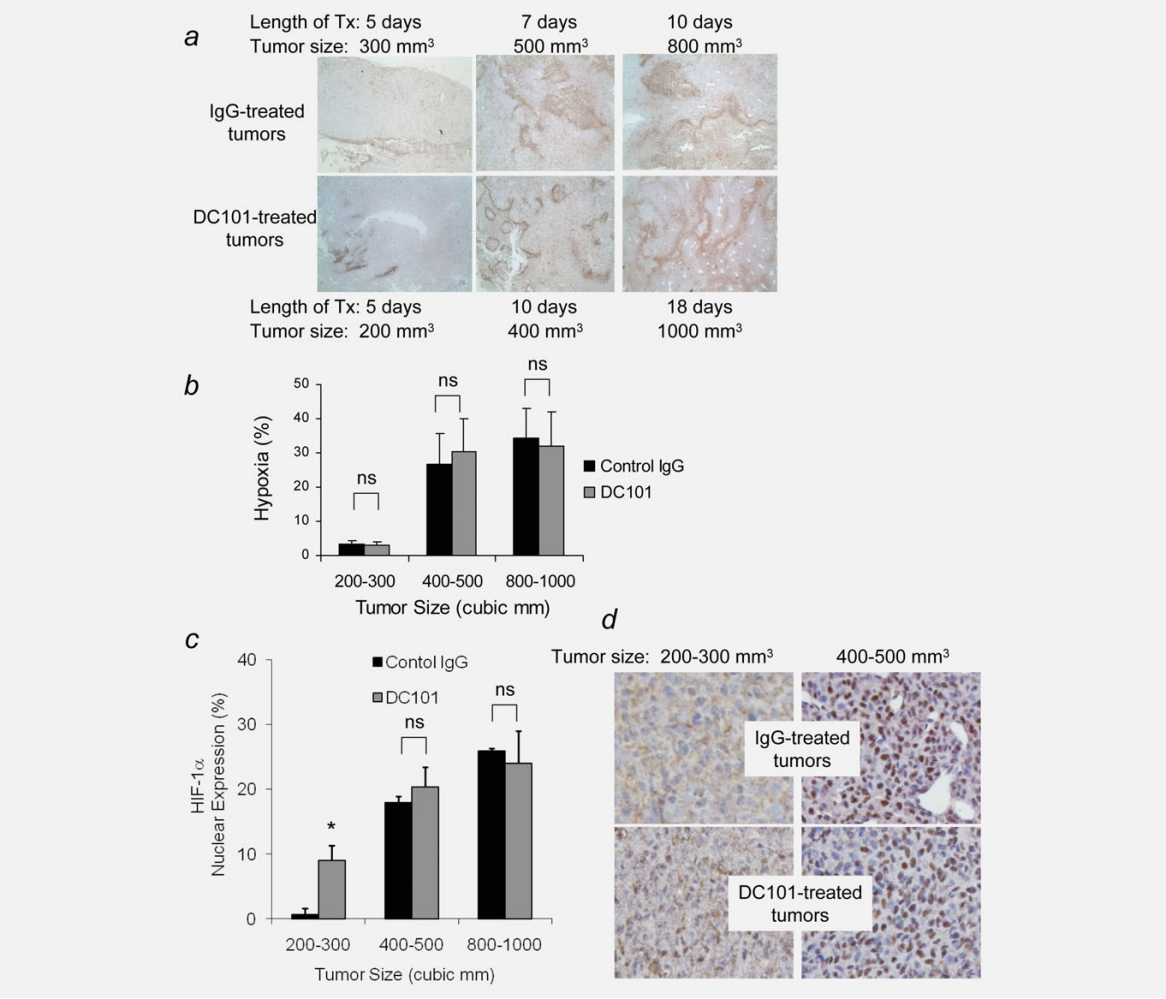
(*a*) Photos of HT1080 sarcoma xenografts treated with control IgG or DC101 and stained for hypoxia using Hypoxyprobe™-1. Length of treatment (Tx) and tumor sizes at time of harvesting are listed. (*b*) Percent hypoxia of H1080 xenografts at various sized tumors after treatment with control IgG or DC101. (*c*) Percentage of nuclei in various sized tumors treated with control IgG or DC101 staining for HIF-1α or HIF-2α. Bars represent standard deviation. *p < 0.05 compared to control IgG group; ns, not significant (*p* > 0.05). (*d*) Photos of HT1080 sarcoma xenografts treated with control IgG or DC101 and stained for hypoxia HIF-1a. Tumor sizes at time of harvesting are listed. [Color figure can be viewed in the online issue, which is available at wileyonlinelibrary.com.]

We examined our sarcoma cell lines for the expression of VEGF receptors 1 and 2 (VEGFR-1 and VEGFR-2) and found little or no expression in any of these cell lines (data not shown). Using HT1080 fibrosarcoma flank tumor xenografts generated in athymic nude mice, we examined the levels of hypoxia and HIF-1α in control tumors and the tumors treated with DC101, an anti-VEGFR-2 antibody. Tumor-bearing mice were treated with DC101 or control IgG once they reached 50 mm^3^ in size. Control tumors took ∼12 days to reach 1,000 mm^3^ (data not shown). When analyzed for the levels of hypoxia using Hypoxyprobe immunohistochemistry, hypoxia levels were found to increase in control tumors as tumors increased from 200 to 500 mm^3^, with no further increase in hypoxia as tumor grew beyond 500 mm^3^ (Fig. 3*a*). HT1080 xenografts treated with DC101 showed delayed tumor growth, with tumors taking about 5–7 days longer to reach 1,000 mm^3^ in size. The levels of hypoxia in HT1080 tumors were not significantly affected by DC101 after controlling for tumor size (*i.e.*, comparing similar-sized tumors in each group). The levels of nuclear HIF-1α correlated with the levels of intratumoral hypoxia, with levels increasing as tumors increased in size from 200 to 500 mm^3^ and then remaining stable (Fig. 3*b*). Of note, DC101 treatment did increase nuclear localization of HIF-1α in small tumors (200–300 mm^3^; Figs. 3*c* and 3*d*). This would suggest that there is no direct correlation between the levels of hypoxia as measured by Hypoxyprobe staining and the stabilization of HIF-1α protein, with the latter possibly being a more sensitive measure of hypoxia. Alternatively, DC101 may have effects on HIF-1α nuclear localization, which are independent of hypoxia levels.

**Figure 4:**
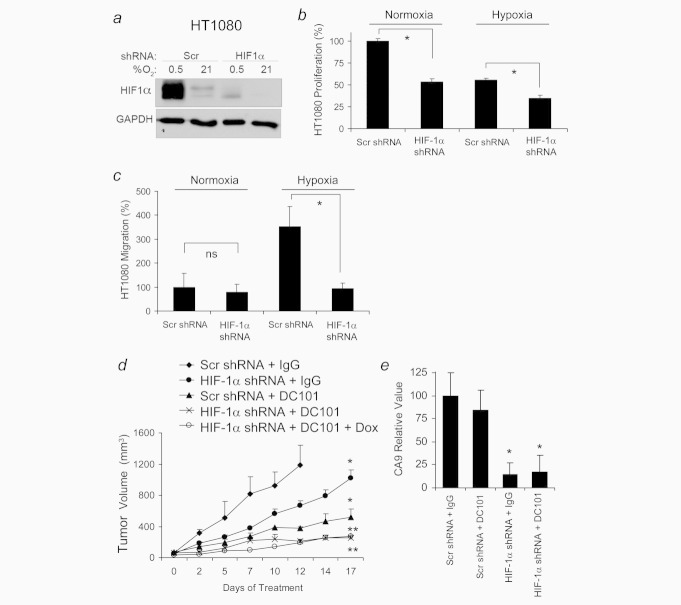
(*a*) Western blot analysis of HIF-1α in HT1080 cells in 21% oxygen (normoxia) and 0.5% oxygen (hypoxia) following treatment with HIF-1α shRNA or scrambled (Scr) control shRNA. GAPDH blot serves as loading control. Proliferation (*b*) and migration (*c*) of HT1080 cells after transduction with HIF-1α shRNA or scrambled (Scr) shRNA. (*d*) Growth of HT1080 cell transduced with HIF-1α shRNA or scrambled (Scr) shRNA following subcutaneous injection in athymic nude mice. Some groups treated with DC101 and IgG were used as antibody treatment control. Metronomic doxorubicin (Dox) was given to one group. (*e*) Relative mRNA levels of *CA9* in HT1080 tumor groups. Bars represent standard deviation. *p < 0.05 compared to control group; ns, not significant (*p* > 0.05); **p < 0.05 compared to Scr shRNA + IgG control group, HIF-1α shRNA + IgG group and Scr shRNA + DC101 group.

To determine the role of HIF-1α in sarcoma cell proliferation and migration *in vitro* and tumor growth *in vivo*, we used shRNA knockdown of *HIF-1α* in HT1080 sarcoma cells that showed strong upregulation of HIF-1α in response to 0.5% hypoxia (Fig. 4*a*). This upregulation of HIF-1α was effectively blocked by shRNA knockdown of *HIF-1α*. shRNA knockdown in HT1080 cells inhibited proliferation under normoxic and hypoxic conditions (Fig. 4*b*) and reduced the migration of these cells under hypoxic conditions (Fig. 4*c*). Following *HIF-1α* knockdown in MS5907 sarcoma cell lines, we found no effect on proliferation; however, we found decreased migration under hypoxic conditions (Supporting Information Figs. S1A–S1C). HT1080 cells with stable knockdown of *HIF-1α* and control HT1080 cells were then grown as flank xenografts with or without treatment with DC101. The knockdown of *HIF-1α* or DC101 treatment inhibited tumor growth; however, the combination of HIF-1α knockdown and DC101 caused the greatest degree of growth inhibition (Fig. 4*d*). We assessed the levels of one HIF-1α target gene, *CA9*, in treated HT1080 tumors by qRT-PCR and found that HIF-1α knockdown did indeed repress *CA9* levels (Fig. 4*e*).

**Figure 5:**
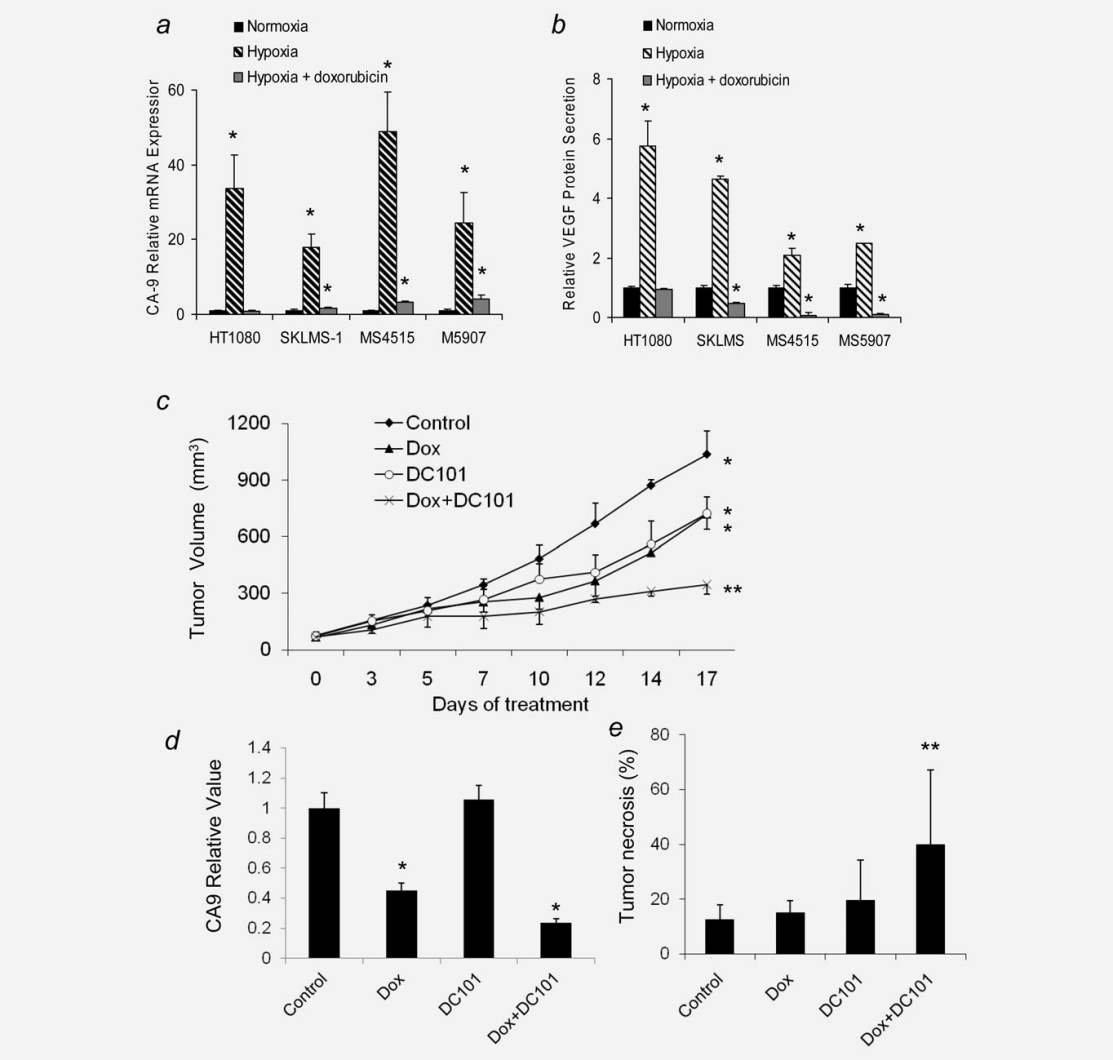
(*a*) Relative mRNA levels of *CA9* or *FOXM1* in human and mouse sarcoma cell lines under normoxia, hypoxia or hypoxia with doxorubicin (10 μM). (*b*) VEGF-A protein secreted into media of human and sarcoma cell lines under normoxia, hypoxia or hypoxia with doxorubicin (10 μM). (*c*) Growth of HT1080 fibrosarcoma xenografts *in vivo*. HT1080 cells were injected subcutaneously, and growth was monitored for 17 days. (*d*) Relative mRNA levels of *CA9* in HT1080 tumors. (*e*) Percent necrosis in HT1080 tumors as determined following H&E staining. For relative values, expression level under normoxia for each cell line or control group assigned a value of 1. Bars represent standard deviation. *p ≤ 0.05 compared to control group; **p < 0.05 compared to control group and to other treatment groups.

Lee *et al*.^17^ found after screening 3,120 drugs from the Johns Hopkins Drug Library that Dox is a potent inhibitor of HIF-1α by blocking HIF-1α binding to DNA. Dox is the most commonly used chemotherapeutic agent for soft tissue sarcomas,^17^ and thus, the use of this agent to block HIF-1α induction of target genes in sarcomas makes therapeutic sense. Dox blocked proliferation of all four sarcoma cell lines and two types of endothelial cells at IC_50_ concentrations of 0.005 to 0.1 μM, with endothelial cells generally being more sensitive to Dox than cancer cell lines (data now shown). As noted earlier for the four sarcoma cell lines, *VEGF-A* and *CA9* were upregulated in hypoxia. For HT1080 cells, *CA9* showed the most hypoxic upregulation of the four genes examined, and low-dose Dox completely potently blocked this hypoxic upregulation (Fig. 5*a*). Low-dose Dox was also able to decrease VEGF-A secretion from all four sarcoma cell lines following hypoxia (Fig. 5*b*). In HT1080 cells, we compared the ability of HIF-1α shRNA and Dox to block induction of VEGF under hypoxic conditions and found that HIF-1α and Dox blocked VEGF secretion equally (Supporting Information Fig. S2). Combining HIF-1α shRNA with Dox had no additive effect (Supporting Information Fig. S2). We next examined the effects of DC101 and/or metronomic Dox on HT1080 xenografts. DC101 or metronomic Dox inhibited tumor growth by 30–31%, and the combination of DC101 or metronomic Dox inhibited tumor growth by 67% (Fig. 5*c*). Tumors were harvested at the end of treatment and examined for the expression of *CA9*. As seen in our *in vitro* studies, HT1080 xenografts exposed to metronomic Dox had significantly lower expression of *CA9* (Fig. 5*d*), and only combination therapy induced significant tumor necrosis (Fig. 5*e*).

**Figure 6:**
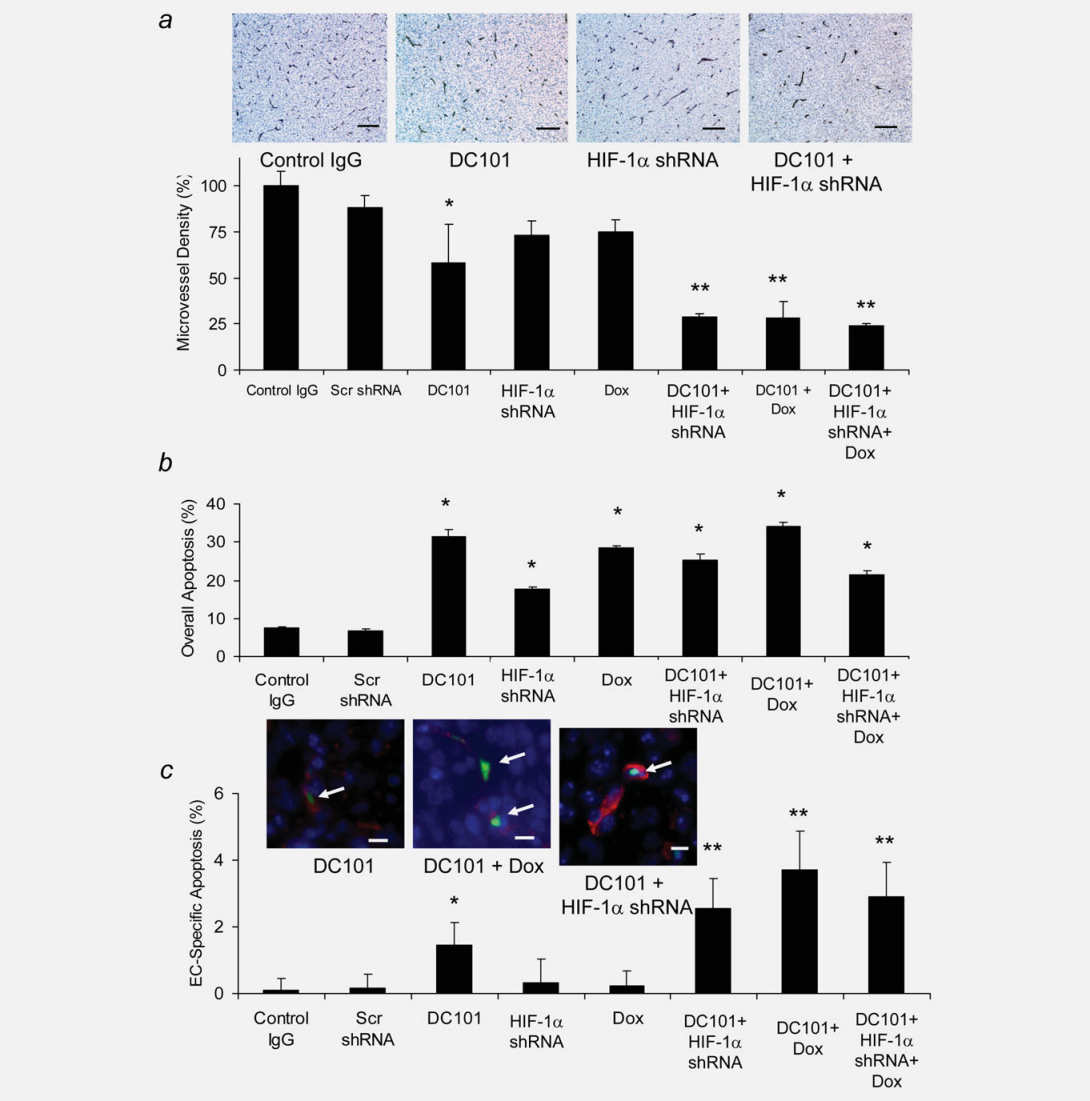
(*a*) Photos of CD31 immunohistochemical staining of HT1080 tumors along with graph showing microvessel density. Scale bar = 100 mm. (*b*) Overall apoptosis in HT1080 tumor groups. (*c*)Photos of coimmunofluorescence for CD31 and TUNEL along with the graph of endothelial cell-specific apoptosis. Scale bar = 7 mm. Arrows point to TUNEL-positive and CD31-positive cells. Bars represent standard deviation. *p ≤ 0.05 compared to control IgG group; **p < 0.05 compared to control IgG and DC101 groups.

We next explored the effects of DC101 along with HIF-1α inhibition (either with HIF-1α shRNA or metronomic Dox) on tumor vasculature. Given the first metronomic Dox likely has off-target effects beyond HIF-1α inhibition, we examined whether adding metronomic Dox to DC101 and HIF-1α knockdown increased HT1080 xenograft tumor growth delay and found that there was no significant additional effect (Fig. 4*d*). Second, we examined MVD and found that DC101 reduced MVD by 42%, HIF-1α shRNA or metronomic Dox by 25–27% and the combination of HIF-1α shRNA or Dox and DC101 by 71–72% (Fig. 6*a*). Overall apoptosis in tumors was increased by 2.3- to 4.5-fold by treatment with DC101 and/or HIF-1α inhibition (Fig. 6*b*), whereas endothelial cell-specific apoptosis was increased at least twofold only with the combination of DC101 and HIF-1α inhibition (Fig. 6*c*). Of note, the addition of Dox to HIF-1α knockdown did not add any further effect on tumor growth inhibition and also did not further decrease MVD or increase endothelial cell-specific apoptosis.

Given the combined VEGF and HIF-1α inhibition appeared to have a synergistic effect on tumor vasculature, we further examined the effect of VEGF-A deprivation plus HIF-1α inhibition on endothelial cells *in vitro*. The removal of VEGF-A from the cell culture media combined with HIF-1α knockdown inhibited the proliferation of HUVECs under both normoxic and hypoxic conditions (Supporting Information Fig. S3A). Similar results were obtained when this experiment was performed with HDMECs (data not shown). We substituted HIF-1α genetic inhibition (*i.e.*, HIF-1α shRNA) with pharmacologic inhibition using low-dose Dox in HDMECs and found a similar synergistic attenuation of proliferation when VEGF-A withdrawal was combined with Dox (Supporting Information Fig. S3B).

## Discussion

Our study was initiated following the examination of correlative science studies from a Phase II clinical trial of bevacizumab and radiation therapy for sarcomas. In this clinical trial, the addition of VEGF-A inhibition to radiation significantly increased the proportion of tumors with a good response to radiation therapy to nearly 50%. Studies of tumor tissue obtained before treatment and during treatment allowed us to ask why the other 50% of tumors were resistant to therapy. In our study, analysis of gene expression microarrays suggested that high expression of HIF-1α and HIF-1α target genes contributed to resistance to the combination of bevacizumab and radiation. Thus, we examined the role of hypoxia and HIF-1α in sarcoma progression as well as the combination of VEGF-A and HIF-1α inhibition in sarcomas. We found that four different sarcoma cells lines upregulate HIF-1α and specific HIF-1α target genes under hypoxic conditions. Genetic deletion of HIF-1α using shRNA or the pharmacologic blockade of HIF-1α binding to target DNA using low-dose Dox act synergistically with VEGF inhibition to suppress the growth of sarcoma xenografts. Our analysis of treated tumors reveals that the one mechanism for this effect is *via* the induction of tumor endothelial cell apoptosis. These findings have significant implications in the future treatment of sarcomas with antiangiogenic therapies.

When antiangiogenic therapies were initially proposed for inhibiting solid tumors, it was thought that such therapies would be less susceptible to resistance given the target was genetically stable tumor endothelial cells as opposed to genetically unstable cancer cells. Now after several years of antiangiogenic therapies being used in patients with solid tumors, oncologists have found that antiangiogenic therapies generally result only in a transitory inhibition or delay in tumor growth with ultimate regrowth of tumors. Mechanisms of resistance to antiangiogenic therapies include upregulation of alternative proangiogenic signals, protection of the tumor vasculature either by recruiting proangiogenic inflammatory cells or by increasing protective pericyte coverage, accentuated invasiveness of tumor cells into local tissue to co-opt normal vasculature and increased metastatic seeding and tumor cell growth in lymph nodes and distant organs.^43^

Hypoxia and HIF-1α are known to play important roles in human sarcomas. We have previously shown that the expression of HIF-1α and 25 other hypoxia-related genes in human sarcomas is highly upregulated compared to normal tissues,^19^ and hypoxia in human sarcomas is associated with a higher risk of recurrence and decreased overall survival.^44^, ^45^ HIF-1α upregulates the expression of VEGF-A in sarcomas,^19^ and circulating levels of VEGF-A are elevated on average tenfold in patients with sarcoma compared to controls.^20^ In our study, we confirmed upregulation of HIF-1α under hypoxic conditions in four different sarcoma cell lines. The hypoxia response in terms of target genes was fairly similar between these sarcoma cell lines with uniform upregulation of *VEGF-A* and *CA9*.

The effect of VEGF inhibition on intratumoral hypoxia and HIF-1α activity may vary depending on the specific tumor type. The administration of anti-VEGF agents can result in reduced vessel irregularity, diameter and permeability and can transiently improve the delivery of oxygen.^27^ However, sustained anti-VEGF therapy can ultimately lead to loss of tumor vessels and increased hypoxia.^28^ In our study, we found that anti-VEGFR-2 therapy with DC101 did not significantly change intratumoral hypoxia when comparing similar-sized tumors; however, DC101 did appear to increase nuclear localization of HIF-1α in small tumors (200–300 mm^3^).

One mechanism by which VEGF inhibition and HIF-1α inhibition synergistically inhibit sarcoma progression is *via* the targeting of the tumor endothelium. Thus, VEGF inhibition likely only inhibits the vascular compartment of tumors and has little or no effect on the cancer cell compartment. HIF-1α, in contrast, is expressed by both endothelial cells and sarcoma cells and may have effects on both cell types. We analyzed these compartments separately both *in vitro* and *in vivo* and found that synergistic effects from combined VEGF inhibition and HIF-1α inhibition on proliferation and apoptosis were only found in the vascular compartment.

Lee *et al*.^17^ screened more than 3,000 drugs from the Johns Hopkins Drug Library and found that Dox at low doses can block HIF-1α binding to DNA. In looking for a clinically applicable inhibitor of HIF-1a for sarcomas, Dox is a very good candidate, given that Dox is already the most commonly used chemotherapeutic drug for sarcomas.^46^ However, the traditional means of delivering Dox, that is, at maximum tolerated doses, focuses on maximizing cancer cell cytotoxicity rather than maximizing HIF-1α blockade. The delivery of Dox at low, continuous doses (*i.e.*, metronomic doses) maximizes HIF-1α blockade,^17^ and this approach has been used in other patients with solid tumors. The administration of metronomic Dox to heavily pretreated patients with metastatic breast cancer resulted in a partial response in 18% of patients and stable disease in 3%.^47^ We found in our study that metronomic Dox can block HIF-1α-mediated upregulation of target genes, and when combined with anti-VEGF therapy has a synergistic effect in blocking sarcoma tumor growth.

There are several limitations to our study. One potential criticism is that metronomic Dox is used as an inhibitor of HIF-1α rather than more specific inhibitor of HIF-1α. Dox is a topoisomerase II inhibitor and a commonly used chemotherapeutic agent. Mechanistically, we confirmed the synergistic effects of HIF-1α and VEGF inhibition on tumor growth and on tumor endothelium by specifically knocking down *HIF-1α* using shRNA. There are several HIF-1α inhibitors that are in various phases of clinical development; however, none are approved for clinical use.^48^ Although Dox clearly has effects other than inhibiting HIF-1α, the primary advantage of using metronomic Dox in our study is that Dox is already clinically approved for the treatment of sarcomas and that findings from our study can be immediately translated into clinical trials combining metronomic Dox with VEGF inhibitors such as bevacizumab.

In conclusion, sarcomas are a heterogeneous group of solid tumors in which hypoxia and HIF-1α activity play varying roles. A strong HIF-1α transcriptional program may be a means of resistance for some sarcomas to anti-VEGF therapy. In addition, HIF-1α inhibition to VEGF inhibition augments destruction of the tumor vasculature in sarcomas, and this strategy should be investigated in clinical trials.
