# Supplementary figures and images for: Overcoming evasive resistance from vascular endothelial growth factor a inhibition in sarcomas by genetic or pharmacologic targeting of hypoxia-inducible factor 1α
Source: Int J Cancer. 2012 Jun 26;132(1):29–41. doi: 10.1002/ijc.27666 (PMC3677782; doi:10.1002/ijc.27666)

A.

MS5907

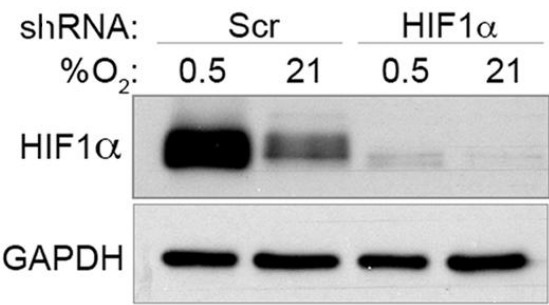

B.

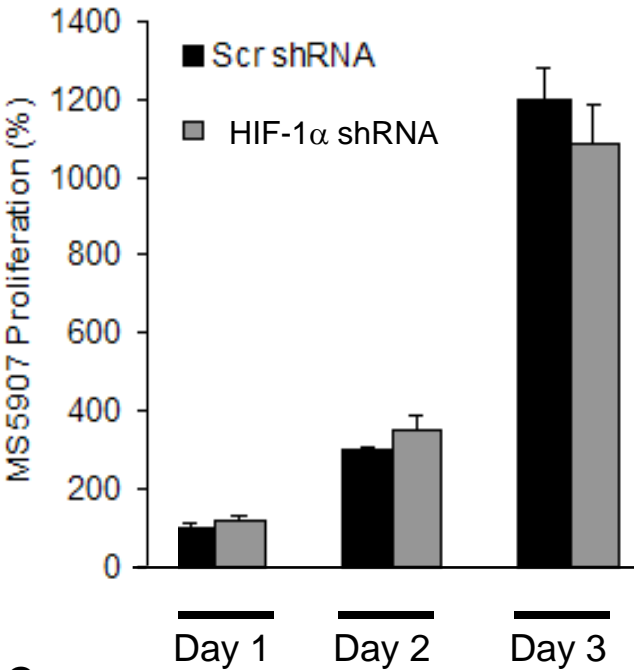

C.

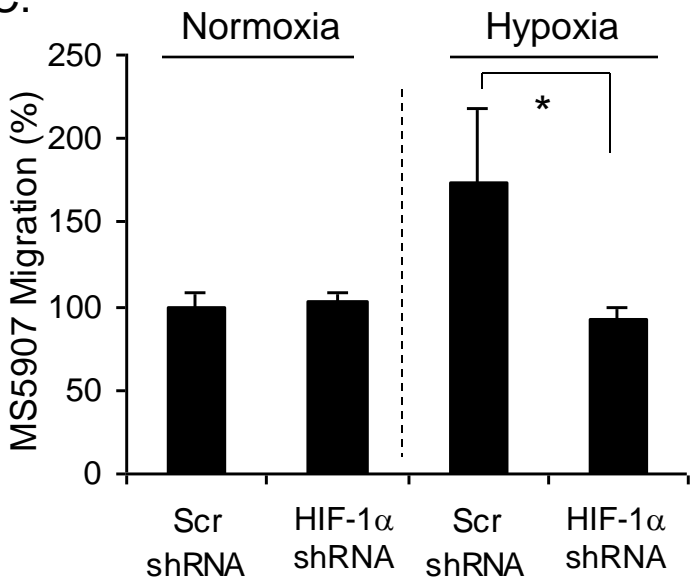

Kim YJ et al. Supplemental Figure 2

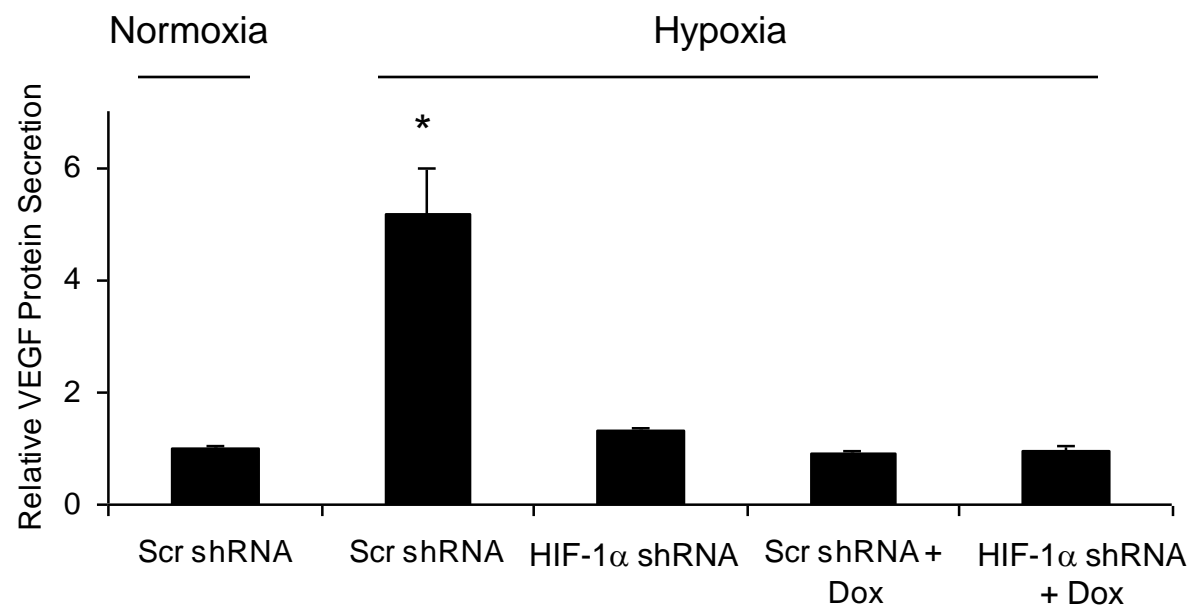

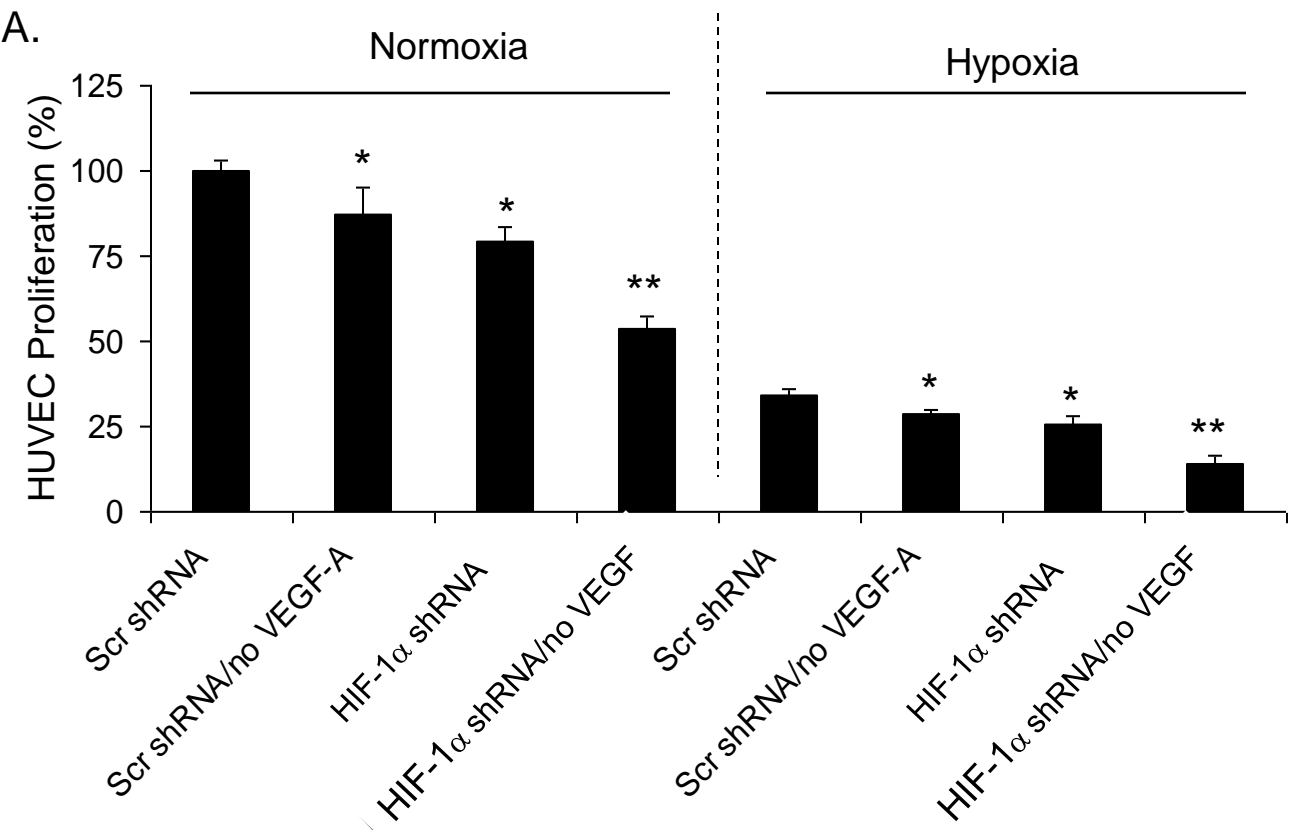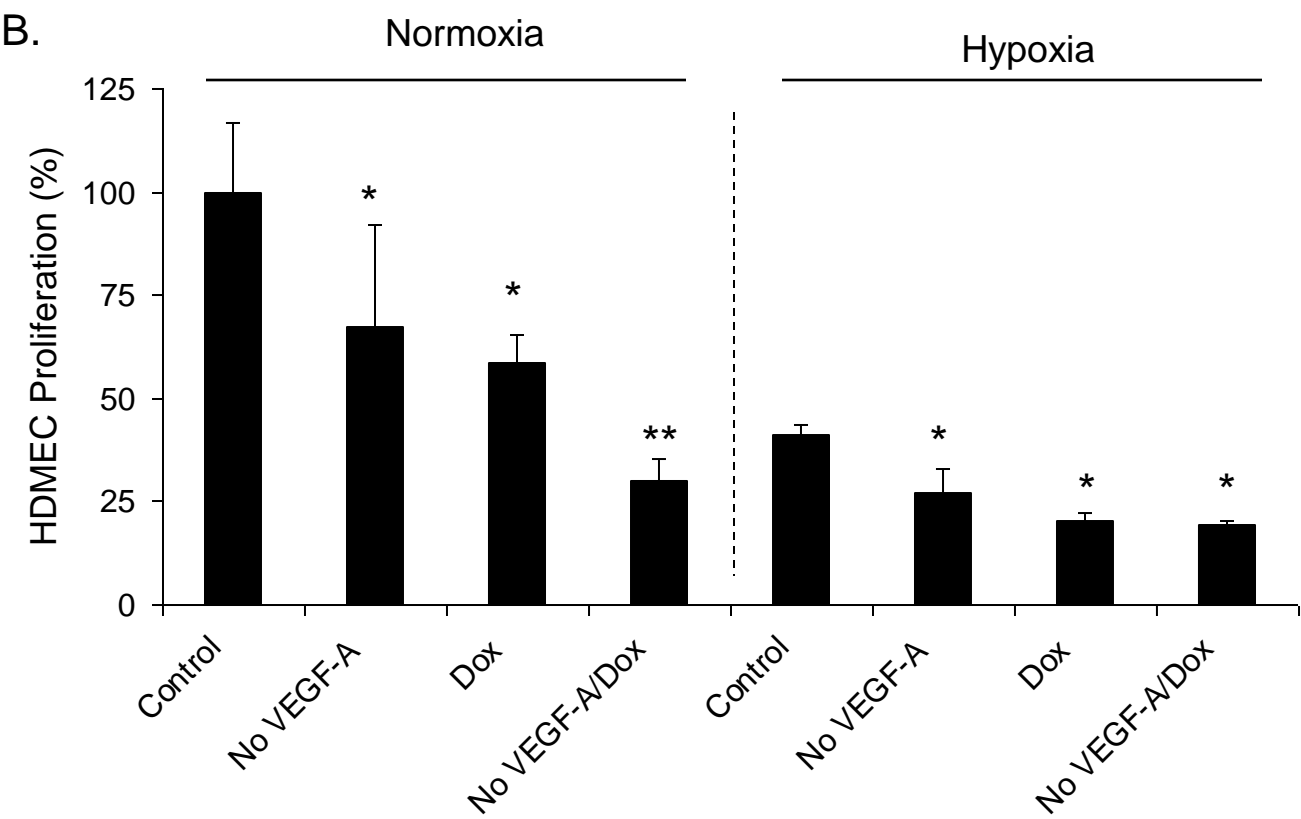

Supplement: Supplementary file 1 [file ijc0132-0029-sd1.pdf]
