# Supplementary material for: Overcoming evasive resistance from vascular endothelial growth factor a inhibition in sarcomas by genetic or pharmacologic targeting of hypoxia-inducible factor 1α
Source: Int J Cancer. 2012 Jun 26;132(1):29–41. doi: 10.1002/ijc.27666 (PMC3677782; doi:10.1002/ijc.27666)
Supplement: Supplementary file 2 [file ijc0132-0029-sd2.doc]

| Up-regulated | GO functions | Genes | Nominal p value |
| --- | --- | --- | --- |
| Poor-responder | ENZYME_LINKED_RECEPTOR_PROTEIN_SIGNALING_PATHWAY | 139 | 0.008069 |
|  | POSITIVE_REGULATION_OF_T_CELL_ACTIVATION | 21 | 0.014388 |
|  | NEGATIVE_REGULATION_OF_SIGNAL_TRANSDUCTION | 36 | 0.018211 |
|  | NEUROPEPTIDE_BINDING | 23 | 0.023632 |
|  | FEEDING_BEHAVIOR | 22 | 0.024856 |
|  | NEUROPEPTIDE_RECEPTOR_ACTIVITY | 22 | 0.025784 |
|  | TRANSMEMBRANE_RECEPTOR_PROTEIN_TYROSINE_KINASE_SIGNALING_PATHWAY | 83 | 0.0368 |
|  | NERVOUS_SYSTEM_DEVELOPMENT | 378 | 0.039565 |
|  | POSITIVE_REGULATION_OF_LYMPHOCYTE_ACTIVATION | 24 | 0.042291 |
|  | CELL_CELL_ADHESION | 85 | 0.066201 |
|  | OXIDOREDUCTASE_ACTIVITY__ACTING_ON_THE_ALDEHYDE_OR_OXO_GROUP_OF_DONORS | 22 | 0.078365 |
|  | RESPONSE_TO_HYPOXIA | 28 | 0.08122 |
|  | CENTRAL_NERVOUS_SYSTEM_DEVELOPMENT | 119 | 0.082243 |
|  | LIPASE_ACTIVITY | 49 | 0.092953 |
| Good-responder | COATED_VESICLE | 46 | 0.018042 |
|  | INTRACELLULAR_TRANSPORT | 274 | 0.049191 |
|  | CELLULAR_MACROMOLECULE_CATABOLIC_PROCESS | 100 | 0.056277 |
|  | CHROMOSOME_SEGREGATION | 32 | 0.056615 |
|  | CELLULAR_LOCALIZATION | 365 | 0.062617 |
|  | ENDOPLASMIC_RETICULUM | 289 | 0.067867 |
|  | PROTEASOME_COMPLEX | 23 | 0.070443 |
|  | ESTABLISHMENT_OF_CELLULAR_LOCALIZATION | 347 | 0.076358 |
|  | MACROMOLECULE_CATABOLIC_PROCESS | 133 | 0.0773 |
|  | PHOSPHOINOSITIDE_BIOSYNTHETIC_PROCESS | 23 | 0.08413 |
|  | VESICLE_MEMBRANE | 29 | 0.086 |
|  | NUCLEOBASE__NUCLEOSIDE__NUCLEOTIDE_AND_NUCLEIC_ACID_TRANSPORT | 31 | 0.089339 |
|  | PROTEIN_PROCESSING | 49 | 0.091265 |
|  | BIOPOLYMER_CATABOLIC_PROCESS | 113 | 0.093561 |
|  | CYTOPLASMIC_VESICLE_MEMBRANE | 27 | 0.09435 |
|  | CYTOPLASMIC_VESICLE_PART | 27 | 0.094576 |
|  | ENDOPLASMIC_RETICULUM_PART | 97 | 0.096291 |
|  | INTEGRAL_TO_ORGANELLE_MEMBRANE | 50 | 0.098771 |

**SUPPLEMENTAL TABLE 1:**

**SUPPLEMENTAL TABLE 2**

**SUPPLEMENTAL TABLE 2 (continued)**
